# Supplementary material for: Glycinergic axonal inhibition subserves acute spatial sensitivity to sudden increases in sound intensity
Source: eLife. 2021 Jun 14;10:e62183. doi: 10.7554/eLife.62183 (PMC8238506; doi:10.7554/eLife.62183)
Supplement: Transparent reporting form [file elife-62183-transrepform.pdf]

eLife's transparent reporting form

We encourage authors to provide detailed information *within their submission* to facilitate the interpretation and replication of experiments. Authors can upload supporting documentation to indicate the use of appropriate reporting guidelines for health-related research (see EQUATOR Network), life science research (see the BioSharing Information Resource), or the ARRIVE guidelines for reporting work involving animal research. Where applicable, authors should refer to any relevant reporting standards documents in this form.

If you have any questions, please consult our Journal Policies and/or contact us: editorial@elifesciences.org.

Sample-size estimation

- You should state whether an appropriate sample size was computed when the study was being designed
- You should state the statistical method of sample size computation and any required assumptions
- If no explicit power analysis was used, you should describe how you decided what sample (replicate) size (number) to use

Please outline where this information can be found within the submission (e.g., sections or figure legends), or explain why this information doesn't apply to your submission:

No sample-size calculations were performed. Selected sample sizes are standard to the field. For all analyses on population data, the number of neurons recorded from is indicated in the figure legends. The number of recorded neurons is high for *in vivo* patch clamping in the brainstem, which are technically challenging recordings.

Replicates

- You should report how often each experiment was performed
- You should include a definition of biological versus technical replication
- The data obtained should be provided and sufficient information should be provided to indicate the number of independent biological and/or technical replicates
- If you encountered any outliers, you should describe how these were handled
- Criteria for exclusion/inclusion of data should be clearly stated
- High-throughput sequence data should be uploaded before submission, with a private link for reviewers provided (these are available from both GEO and ArrayExpress)

Please outline where this information can be found within the submission (e.g., sections or figure legends), or explain why this information doesn't apply to your submission:

Throughout the manuscript a clear distinction is made between number of neurons (biological replicates) and number of repetitions within a neuron (technical replicates). The number of neurons for which *in vivo* recordings to clicks were obtained are stated in the first paragraph of results. All neurons for which *in vitro* recordings were obtained are reported in Figure 4 and Figure 4-figure supplement 1. For every population analysis it is indicated in how many neurons the respective analysis was done (Results, Figure legends). The number of repeated trials in acoustic responses is indicated in Methods. As specified in Methods, neurons recorded from were classified as MSO neurons, principal LSO neurons or non-principal LSO neurons using the same criteria as in our earlier work (Franken et al., 2018; Franken et al., 2015). As specified in Methods, one outlier LSO cell and two outlier MSO cells were excluded from the calculation of mean +/- SEM of series resistance.

Statistical reporting

- Statistical analysis methods should be described and justified
- Raw data should be presented in figures whenever informative to do so (typically when N per group is less than 10)
- For each experiment, you should identify the statistical tests used, exact values of N, definitions of center, methods of multiple test correction, and dispersion and precision measures (e.g., mean, median, SD, SEM, confidence intervals; and, for the major substantive results, a measure of effect size (e.g., Pearson's r, Cohen's d)
- Report exact p-values wherever possible alongside the summary statistics and 95% confidence intervals. These should be reported for all key questions and not only when the p-value is less than 0.05.

Please outline where this information can be found within the submission (e.g., sections or figure legends), or explain why this information doesn't apply to your submission:

Statistical analysis methods are specified in Methods. Exact p-values are reported throughout the manuscript, as well as precision and dispersion values, the statistical test used, the value of the statistic with exact values of *N*, and measures of effect size when relevant.

(For large datasets, or papers with a very large number of statistical tests, you may upload a single table file with tests, Ns, etc., with reference to sections in the manuscript.)

Group allocation

- Indicate how samples were allocated into experimental groups (in the case of clinical studies, please specify allocation to treatment method); if randomization was used, please also state if restricted randomization was applied
- Indicate if masking was used during group allocation, data collection and/or data analysis

Please outline where this information can be found within the submission (e.g., sections or figure legends), or explain why this information doesn't apply to your submission:

For the *in vivo* experiments, LSO or MSO recordings were obtained in an animal depending on the success of gigaseal formation. Because these nuclei are close together, one cannot reliably aim for one or the other nucleus. We used the same stimuli when recording from MSO and LSO neurons. A possible covariate is frequency tuning of neurons (characteristic frequency, CF), which is reported in several figures, showing that the range of CF covered in the LSO and MSO data is similar, as stated in Methods. For the *in vitro* slice recordings, formal randomization between simulated excitation and inhibition is not possible. Neurons were recorded in a region of the nucleus that corresponded to the *in vivo* recordings, and only selected based on health criteria.
Masking is not relevant to the *in vivo* data, because there are no treatment groups.
Masking is not possible for the *in vitro* recordings, not during the experiment and not for the analysis because of the electrical artefacts caused by electric shock stimulation.
Masking cannot be used for descriptive imaging data that do not compare groups.

Additional data files ("source data")

- We encourage you to upload relevant additional data files, such as numerical data that are represented as a graph in a figure, or as a summary table

eLife Sciences Publications, Ltd is a limited liability non-profit non-stock corporation incorporated in the State of Delaware, USA, with company number 5030732, and is registered in the UK with company number FC030576 and branch number BR015634 at the address Westbrook Centre, Milton Road Cambridge CB4 1YG, UK | March 2019

- Where provided, these should be in the most useful format, and they can be uploaded as "Source data" files linked to a main figure or table
- Include model definition files including the full list of parameters used
- Include code used for data analysis (e.g., R, MatLab)
- Avoid stating that data files are "available upon request"

Please indicate the figures or tables for which source data files have been provided:

Source data files have been provided for numerical data represented as graphs in Figure 1 and supplements 1-5, Figure 2 and supplement 1, Figure 3 and supplement 1, Figure 4 and supplement 1, Figure 7 and supplement 2 and Figure 8 and supplements 1-2.
